# Effects of short‐course preoperative endocrine therapy on tumour morphology and immunohistochemical profile in oestrogen receptor‐positive, HER2‐negative breast cancer

**DOI:** 10.1111/his.70203

**Published:** 2026-06-18

**Authors:** Claudia Grosse, Alexandra Grosse, Petar Noack, Caroline Ines Preuss, Heike Kathleen Schwarz, Bruno Schneeweiss, Thomas Gitter, Peter Schrenk, Rupert Langer

**Affiliations:** ^1^ Department of Clinical Pathology and Molecular Pathology Johannes Kepler University Linz, Kepler University Hospital GmbH Linz Austria; ^2^ Pathology Medica Enge AG Zurich Switzerland; ^3^ Department of Gynaecology, Obstetrics and Gynaecological Endocrinology Johannes Kepler University Linz, Kepler University Hospital GmbH Linz Austria; ^4^ Department of Haematology and Oncology Johannes Kepler University Linz, Kepler University Hospital GmbH Linz Austria; ^5^ Central Radiology Institute Johannes Kepler University Linz, Kepler University Hospital GmbH Linz Austria; ^6^ Department of General and Visceral Surgery Johannes Kepler University Linz, Kepler University Hospital GmbH Linz Austria

**Keywords:** breast cancer, Ki67, MammaPrint, preoperative endocrine therapy, progesterone receptor, tumour grading

## Abstract

**Aims:**

Short‐term preoperative endocrine therapy (ET) is increasingly used in oestrogen receptor (ER)‐positive, HER2‐negative breast cancer as a functional test of endocrine sensitivity. We aimed to characterise histomorphological and immunophenotypic changes following preoperative ET and to identify predictors of endocrine response, defined as post‐treatment Ki67 ≤ 10%.

**Methods and results:**

In this retrospective single‐centre study, 180 patients treated with short‐course preoperative ET (median duration 29 days) were compared with 151 patients undergoing primary surgery without ET. Paired biopsy and resection specimens were assessed for histological features, stromal proportion, stromal tumour‐infiltrating lymphocytes (strTILs) and expression of ER, progesterone receptor (PR), HER2 and Ki67. Genomic risk was determined using the MammaPrint assay. Preoperative ET was associated with a significant reduction in tumour proliferation, with 73.9% of cases showing post‐treatment Ki67 ≤ 10% compared with none in controls (*P* < 0.001). Histological grade decreased in 36.7% of ET‐treated tumours versus 7.9% of controls (*P* < 0.001), predominantly reflecting reduced mitotic activity. ER expression remained stable, whereas PR expression decreased more frequently following ET (*P* < 0.001) and was independently associated with Ki67‐defined response. HER2‐low status was more frequently observed after ET (*P* < 0.001), but HER2 expression and microenvironmental parameters, including strTILs, were not associated with response. High genomic risk was independently associated with a lower likelihood of achieving post‐treatment Ki67 ≤ 10% (*P* < 0.001).

**Conclusions:**

Short‐course preoperative ET induces rapid and reproducible morphological and immunophenotypic changes in ER‐positive, HER2‐negative breast cancer. Ki67‐defined response is associated with genomic risk and PR expression, whereas microenvironmental features appear to have limited predictive value.

## Graphical abstract

Short‐course preoperative endocrine therapy induces histopathological and immunophenotypic changes in oestrogen receptor‐positive, HER2‐negative breast cancer. Proliferative response is independently associated with baseline genomic risk (MammaPrint) and progesterone receptor expression.

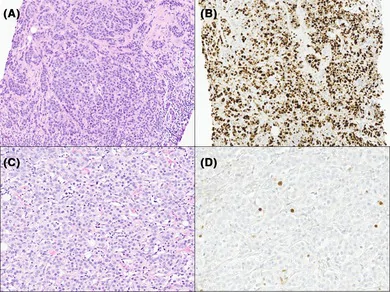

**Abbreviations**
AI: aromatase inhibitor
BC: breast cancer
CI: confidence interval
ER: oestrogen receptor
ET: endocrine therapy
HR: hormone receptor
ISH: in situ hybridisation
NACT: neoadjuvant chemotherapy
NAET: neoadjuvant endocrine therapy
OR: odds ratio
PR: progesterone receptor
SP: stromal proportion
strTIL: stromal tumour‐infiltrating lymphocyte
TSR: tumour–stroma ratio

## Introduction

Hormone receptor (HR)–positive, HER2‐negative breast cancer (BC), defined by immunohistochemical expression of oestrogen receptor (ER) and/or progesterone receptor (PR) in the absence of HER2 overexpression or gene amplification, represents the most common intrinsic subtype, accounting for more than 65% of invasive breast carcinomas.^1^, ^2^ Endocrine therapy is a cornerstone of treatment in this disease. Beyond its established adjuvant role, neoadjuvant endocrine therapy (NAET), typically administered over several months, has emerged as an effective alternative to neoadjuvant chemotherapy (NACT), particularly in patients with strongly hormone‐dependent tumours, where it may facilitate tumour downsizing and breast‐conserving surgery.^3^, ^4^ During the coronavirus disease 2019 (COVID‐19) pandemic, short‐course NAET was increasingly adopted as a bridging strategy to safely defer surgery in selected low‐risk patients because of constrained healthcare resources.^4^, ^5^, ^6^, ^7^, ^8^ In these settings, treatment response has traditionally been assessed by reductions in tumour size or cellularity, and histopathological studies have described decreased tumour cellularity, stromal fibrosis and architectural remodelling following endocrine exposure.^5^, ^9^, ^10^, ^11^, ^12^ More recently, however, the focus of perioperative endocrine studies has shifted from morphological response toward early biological response, particularly changes in tumour proliferation as assessed by Ki67. In this context, short‐course preoperative endocrine therapy (ET) has increasingly been used as a window‐of‐opportunity approach to assess endocrine sensitivity in vivo. Perioperative trials such as IMPACT and POETIC demonstrated that suppression of Ki67 after only 2–4 weeks of ET represents a robust surrogate marker of endocrine sensitivity and is strongly associated with long‐term clinical outcomes.^13^, ^14^ Building on this concept, the WSG‐ADAPT HR+/HER2− trial established a response‐adapted strategy integrating genomic risk assessment with early Ki67 dynamics, using a post‐treatment threshold of ≤10% to define endocrine response.^15^ This approach identifies patients with favourable biology who may safely omit chemotherapy and supports the concept that short‐course preoperative ET primarily functions as an in vivo assay of endocrine responsiveness rather than a cytoreductive intervention.^13^, ^15^, ^16^, ^17^, ^18^

In parallel, genomic assays such as the 70‐gene signature (MammaPrint), as validated in the MINDACT trial, provide robust prognostic information and identify patients with excellent outcomes despite omission of chemotherapy.^19^, ^20^, ^21^, ^22^ Genomic risk is closely linked to endocrine sensitivity, with low‐risk tumours characterised by lower proliferative activity and greater dependence on ER signalling, consistent with a luminal A‐like phenotype and enhanced responsiveness to ET.^19^, ^20^, ^21^ Despite these advances, the histomorphological and immunophenotypic correlates of early endocrine response following short‐course preoperative endocrine induction therapy remain poorly defined.

This study aimed to characterise histomorphological and immunophenotypic changes associated with short‐course preoperative endocrine induction therapy using paired pre‐ and post‐treatment tumour samples in comparison with a contemporaneous control cohort. In addition, we sought to identify clinicopathological and genomic predictors of endocrine response, defined as post‐treatment Ki67 ≤ 10%.

## Patients and Methods

### Study Cohort

This retrospective study included consecutive female patients with stage I‐IIIB ER‐positive, HER2‐negative BC and baseline Ki67 > 10% treated with preoperative ET for approximately 4 weeks between January 2023 and May 2024. Treatment was administered according to national guidelines and institutional protocols. Patients with prior BC, previous ET, NACT treatment or no subsequent surgery were excluded. A contemporaneous untreated control cohort was included for comparison, comprising consecutive patients with non‐metastatic ER‐positive, HER2‐negative BC and baseline Ki67 > 10% who underwent primary surgery without preoperative ET and for whom paired specimens were available. The study was approved by the Ethics Committee of Johannes Kepler University Linz, Austria (IRB 1167/2024).

### Data Collection and Pathological Assessment

Clinical data, including age at diagnosis, duration and regimen of ET, menopausal status, surgical procedure and clinical stage, were retrieved from the institutional database. Menopausal status was determined based on age, menstrual history and hormonal assessment where indicated. Patients aged >60 years were classified as postmenopausal. In younger patients, classification was based on menstrual history, while serum hormone levels were evaluated in cases of prior hysterectomy or unclear clinical history. The following histopathological and immunohistochemical parameters were independently evaluated on paired biopsy and resection specimens by two experienced breast pathologists, with consensus review: histological subtype, tumour grade, central scarring, stromal proportion (SP), stromal tumour‐infiltrating lymphocytes (strTILs), ER and PR status (including Allred scores), HER2 status and Ki67. Ki67 was assessed by visual estimation as the percentage of positively stained tumour cell nuclei across the entire tumour section, including hotspot regions, in accordance with the updated recommendations from the International Ki67 in Breast Cancer Working Group.^23^, ^24^ ET response was defined as post‐treatment Ki67 ≤ 10%, consistent with response‐adapted clinical trial designs.^15^ Tumour grading was performed according to the Nottingham grading system (Elston–Ellis modification of the Scarff–Bloom–Richardson system), incorporating tubule formation, nuclear pleomorphism and mitotic count.^25^ Changes in overall tumour grade were defined as a shift of at least one grade between pre‐ and post‐treatment specimens. Individual grading components (tubule formation, nuclear pleomorphism and mitotic count) were analysed separately, and changes in these components were defined as a shift of at least one score level. SP, a parameter conceptually related to the tumour–stroma ratio (TSR), was assessed on haematoxylin and eosin–stained sections as the percentage of stromal area relative to the total tumour area. Evaluation was performed in the most stroma‐rich region of the invasive tumour using a × 10 objective field, ensuring tumour cells were present at all borders of the field of view. Areas containing necrosis, adipose tissue, in situ carcinoma and artefacts were excluded from analysis. SP was recorded in 10% increments and analysed both as a continuous variable and as a categorical variable (stroma‐low, ≤50%; stroma‐high, >50%).^26^, ^27^, ^28^, ^29^ StrTILs were assessed on haematoxylin and eosin–stained sections within the stromal compartment of the invasive tumour in accordance with the recommendations of the International Immuno‐Oncology Biomarker Working Group.^30^, ^31^ StrTILs were recorded as a continuous variable and categorised as low (0%–9%), intermediate (10%–59%) or high (60%–100%).^32^

### Immunohistochemical and Genomic Analysis

Immunohistochemistry was performed using a Ventana Benchmark ULTRA automated immunostainer (Roche Tissue Diagnostics, Tucson, AZ, USA). ER, PR and Ki67 were assessed using validated antibodies (ER clone SP1, PR clone 1E2, Ki67 clone 30–9; Roche/Ventana), following antigen retrieval with Cell Conditioning 1 (36 min at 95°C). HER2 status was determined according to standardised immunohistochemistry and, where appropriate, in situ hybridisation (ISH), in accordance with ASCO/CAP guidelines.^33^ HER2 immunohistochemistry was performed using the rabbit monoclonal anti‐HER2 antibody (clone 4B5; Roche/Ventana). ISH was carried out using the VENTANA HER2 Dual ISH DNA Probe Cocktail assay on 4‐μm formalin‐fixed paraffin‐embedded sections. Tumours were classified as HER2‐zero (score 0 or ultra‐low) or HER2‐low (score 1+ or 2+/ISH‐negative) according to contemporary clinical definitions.^34^, ^35^

In the preoperative ET cohort, genomic risk assessment was conducted on biopsy specimens using the MammaPrint assay (Agendia, Amsterdam, The Netherlands); patients were classified as low genomic risk (MammaPrint index ≥0) and high genomic risk (MammaPrint index <0) based on the risk of distant recurrence at 5 years and at 10 years.^36^ Clinical risk was determined using a modified Adjuvant! Online algorithm in accordance with MINDACT criteria,^19^ and patients were stratified into combined clinical/genomic risk groups.

### Statistical Analysis

Continuous variables are presented as mean ± standard deviation, and categorical variables as frequencies and percentages. Group comparisons were performed using the chi‐square test or Fisher's exact test for categorical variables and the Student's *t*‐test for continuous variables. Correlations between Ki67 and other continuous variables were assessed using Pearson's correlation coefficient. Univariate and multivariate logistic regression analyses were performed to identify factors associated with Ki67‐defined response to preoperative ET. Only pretreatment variables were included to identify true baseline predictors. Post‐treatment variables and treatment‐induced changes were excluded because these parameters are influenced by therapy and are directly related to the definition of response. Variables with *P* < 0.05 in univariate analysis were entered into the multivariate model. Odds ratios (ORs) with 95% confidence intervals (CIs) were calculated. All tests were two‐sided, and *P* < 0.05 was considered statistically significant. Statistical analyses were performed using SPSS version 28.0 (IBM Corp., Armonk, NY, USA).

## Results

### Patient and Tumour Characteristics

The study included 180 patients treated with preoperative ET and 151 controls. Clinicopathological and immunohistochemical characteristics are summarised in Table 1. The median age at diagnosis was 61 years (range, 29–90 years). Tumours were predominantly invasive carcinoma of no special type and were mainly of pathological stage T1‐T2. In the ET cohort, treatment consisted predominantly of aromatase inhibitors (AIs) (97.2%; letrozole 46.7%, anastrozole 50.6%), including 19 (10.6%) patients receiving letrozole plus GnRH analogues and 18 (10.0%) receiving anastrozole plus GnRH analogues. A minority of patients (2.8%) received tamoxifen in combination with GnRH analogues. The median duration of ET was 29 days (range, 12–38 days), with 43.3% of patients treated for ≤28 days and 56.7% for >28 days. Baseline tumour characteristics, including Ki67, were broadly comparable between the ET and control cohorts. Genomic risk assessment using MammaPrint was available for 75.6% of ET‐treated patients, of whom 47.8% were classified as low genomic risk and 52.2% as high genomic risk.

**Table 1:** Clinicopathological, immunohistochemical and genomic features of the study cohort

Variable	ET (*N* = 180)	Control (*N* = 151)	*P*‐value	ET responders (*N* = 133)	ET non‐responders (*N* = 47)	*P‐*value
	*N* (%)	*N* (%)		*N* (%)	*N* (%)	
Age (years)						
≤50	38 (21.1)	33 (21.9)	0.870	34 (25.6)	4 (8.5)	**0.014**
>50	142 (78.9)	118 (78.1)		99 (74.4)	43 (91.5)	
Menopausal status						
Peri‐/postmenopausal	144 (80.0)	123 (81.5)	0.738	100 (75.2)	44 (93.6)	**0.007**
Premenopausal	36 (20.0)	28 (18.5)		33 (24.8)	3 (6.4)	
cT stage						
T1	121 (67.2)	90 (59.6)	0.055	92 (69.2)	29 (61.7)	0.391
T2	57 (31.7)	53 (35.1)		39 (29.3)	18 (38.3)	
T3/T4	2 (1.1)	8 (5.3)		2 (1.5)	0 (0.0)	
pT stage						
T1	114 (63.3)	86 (57.0)	0.137	87 (65.4)	27 (57.4)	0.294
T2	63 (35.0)	57 (37.7)		43 (32.3)	20 (42.6)	
T3/T4	3 (1.7)	8 (5.3)		3 (2.3)	0 (0.0)	
Tumour size (mm)						
<20	113 (62.8)	84 (55.6)	0.187	86 (64.7)	27 (57.4)	0.379
≥20	67 (37.2)	67 (44.4)		47 (35.3)	20 (42.6)	
Type of surgery						
BCT	145 (80.6)	110 (72.8)	0.097	112 (84.2)	33 (70.2)	**0.037**
Mastectomy	35 (19.4)	41 (27.2)		21 (15.8)	14 (29.8)	
LN surgery (missing: 6/2)^a^						
SNB	128 (73.6)	86 (57.7)	**0.003**	97 (75.2)	31 (68.9)	0.409
SNB + LND	46 (26.4)	63 (42.3)		32 (24.8)	14 (31.1)	
pN stage (missing: 6/2)^a^						
N0	107 (61.5)	75 (50.3)	**0.018**	78 (60.5)	29 (64.4)	0.855
N1	60 (34.5)	57 (38.3)		46 (35.7)	14 (31.1)	
N2/3	7 (4.0)	17 (11.4)		5 (3.9)	2 (4.4)	
LN status (missing: 6/2)^a^						
Positive	67 (38.5)	74 (49.7)	**0.044**	51 (39.5)	16 (35.6)	0.637
Negative	107 (61.5)	75 (50.3)		78 (60.5)	29 (64.4)	
Subtype baseline						
No special type	134 (74.4)	122 (80.8)	0.261	99 (74.4)	35 (74.5)	0.505
Lobular carcinoma	24 (13.3)	12 (7.9)		16 (12.0)	8 (17.0)	
Other	22 (12.2)	17 (11.3)		18 (13.5)	4 (8.5)	
Subtype post						
No special type	133 (73.9)	119 (78.8)	0.292	98 (73.7)	35 (74.5)	0.459
Lobular carcinoma	24 (13.3)	12 (7.9)		16 (12.0)	8 (17.0)	
Other	23 (12.8)	20 (13.2)		19 (14.3)	4 (8.5)	
Grade baseline						
G1	24 (13.3)	22 (14.6)	0.731	21 (15.8)	3 (6.4)	**0.027**
G2	132 (73.3)	105 (69.5)		99 (74.4)	33 (70.2)	
G3	24 (13.3)	24 (15.9)		13 (9.8)	11 (23.4)	
Grade post						
G1	77 (42.8)	21 (13.9)	**<0.001**	70 (52.6)	7 (14.9)	**<0.001**
G2	93 (51.7)	97 (64.2)		63 (47.4)	30 (63.8)	
G3	10 (5.6)	33 (21.9)		0 (0.0)	10 (21.3)	
SP baseline (%)						
Mean, median	58.7 ± 16.6, 60.0	58.3 ± 18.3, 60.0	0.867	60.8 ± 14.0	52.8 ± 21.6	**0.021**
Stroma‐low (≤50)	60 (33.3)	56 (37.1)	0.476	37 (27.8)	23 (48.9)	**0.008**
Stroma‐high (>50)	120 (66.7)	95 (62.9)		96 (72.2)	24 (51.1)	
SP post (%)						
Mean, median	57.5 ± 16.7, 60.0	55.4 ± 17.3, 60.0	0.255	59.5 ± 15.7	51.9 ± 18.4	**0.007**
Stroma‐low (≤50)	62 (34.4)	51 (33.8)	0.898	40 (30.1)	22 (46.8)	**0.038**
Stroma‐high (>50)	118 (65.6)	100 (66.2)		93 (69.9)	25 (53.2)	
Central scar resection						
Yes	38 (21.1)	33 (21.9)	0.870	22 (16.5)	16 (34.0)	**0.011**
No	142 (78.9)	118 (78.1)		111 (83.5)	31 (66.0)	
TILs baseline (%)						
Mean, median	3.7 ± 7.3, 1.0	2.6 ± 5.5, 0.5	0.108	3.4 ± 5.6	4.9 ± 10.9	0.365
Low TILs (0–9)	168 (93.3)	143 (94.7)	0.603	125 (94.0)	43 (91.5)	0.555
Intermediate TILs (10–59)	12 (6.7)	8 (5.3)		8 (6.0)	4 (8.5)	
TILs post						
Mean, median	4.1 ± 7.5, 1.0	3.6 ± 6.0, 1.0	0.501	4.2 ± 7.0	4.0 ± 8.8	0.913
Low TILs (0%–9%)	161 (89.4)	134 (88.7)	0.838	118 (88.7)	43 (91.5)	0.596
Intermediate TILs (10%–59%)	19 (10.6)	17 (11.3)		15 (11.3)	4 (8.5)	
Ki67 baseline (%)						
Mean, median	22.9 ± 10.5, 20.0	23.6 ± 9.4, 22.0	0.524	21.4 ± 8.9, 18.0	27.0 ± 13.4, 23.0	**0.009**
<20	85 (47.2)	56 (37.1)	0.063	69 (51.9)	16 (34.0)	**0.035**
≥20	95 (52.8)	95 (62.9)		64 (48.1)	31 (66.0)	
Ki67 post (%)						
Mean, median	9.1 ± 8.8, 6.0	23.8 ± 9.2, 23.0	**<0.001**	5.1 ± 2.3, 5.0	20.3 ± 10.6, 18.0	**<0.001**
<10	133 (73.9)	0 (0.0)	**<0.001**	133 (100.0)	0 (0.0)	‐
≥10	47 (26.1)	151 (100.0)		0 (0.0)	47 (100.0)	
ER status baseline/post						
Positive	180 (100.0)	151 (100.0)	‐	133 (100.0)	47 (100.0)	‐
Negative	0 (0.0)	0 (0.0)		0 (0.0)	0 (0.0)	
PR expression baseline (%)						
≥50	49 (27.2)	36 (23.8)	0.483	105 (78.9)	26 (55.3)	**0.002**
<50	131 (72.8)	115 (76.2)		28 (21.1)	21 (44.7)	
PR status baseline						
Positive	164 (91.1)	146 (96.7)	**0.038**	126 (94.7)	38 (80.9)	**0.004**
Negative	16 (8.9)	5 (3.3)		7 (5.3)	9 (19.1)	
PR status post						
Positive	148 (82.2)	147 (97.4)	**<0.001**	113 (85.0)	35 (74.5)	0.106
Negative	32 (17.8)	4 (2.6)		20 (15.0)	12 (25.5)	
HER2 score baseline						
Score 0	17 (9.4)	20 (13.2)	0.366	12 (9.0)	5 (10.6)	0.828
Score 1+	72 (40.0)	51 (33.8)		52 (39.1)	20 (42.6)	
Score 2+	91 (50.6)	80 (53.0)		69 (51.9)	22 (46.8)	
HER2 score post						
Score 0	10 (5.6)	29 (19.2)	**<0.001**	6 (4.5)	4 (8.5)	0.532
Score1+	43 (23.9)	52 (34.4)		31 (23.3)	12 (25.5)	
Score 2+	127 (70.6)	70 (46.4)		96 (72.2)	31 (66.0)	
MammaPrint baseline^b^						
Low risk	‐	‐	‐	57 (57.0)	8 (22.2)	**<0.001**
High risk	‐	‐		43 (43.0)	28 (77.8)	
MammaPrint Index baseline^b^						
Mean	‐	‐	‐	0.048 ± 0.246	−0.160 ± 0.304	**<0.001**
Clinical/genomic risk group						
c‐low/g‐low	‐	‐	‐	27 (27.0)	5 (13.9)	**0.004**
c‐high/g‐high	‐	‐		26 (26.0)	16 (44.4)	
c‐high/g‐low	‐	‐		30 (30.0)	3 (8.3)	
c‐low/g‐high	‐	‐		17 (17.0)	12 (33.3)	

ER, oestrogen receptor; ET, preoperative endocrine therapy; LND, lymph node dissection; LN, lymph node; post, postsurgical/post‐treatment; PR, progesterone receptor; SNB, sentinel lymph node biopsy; SP, stromal proportion; TILs, tumour‐infiltrating lymphocytes. Values in bold indicate statistical significance (*P* < 0.05).

^a^ No lymph nodes were removed in 6 ET and 2 control patients; all were cN0.

^b^ MammaPrint was available in 136 (75.6%) ET‐treated patients.

### Endocrine Response

**Figure 1:**
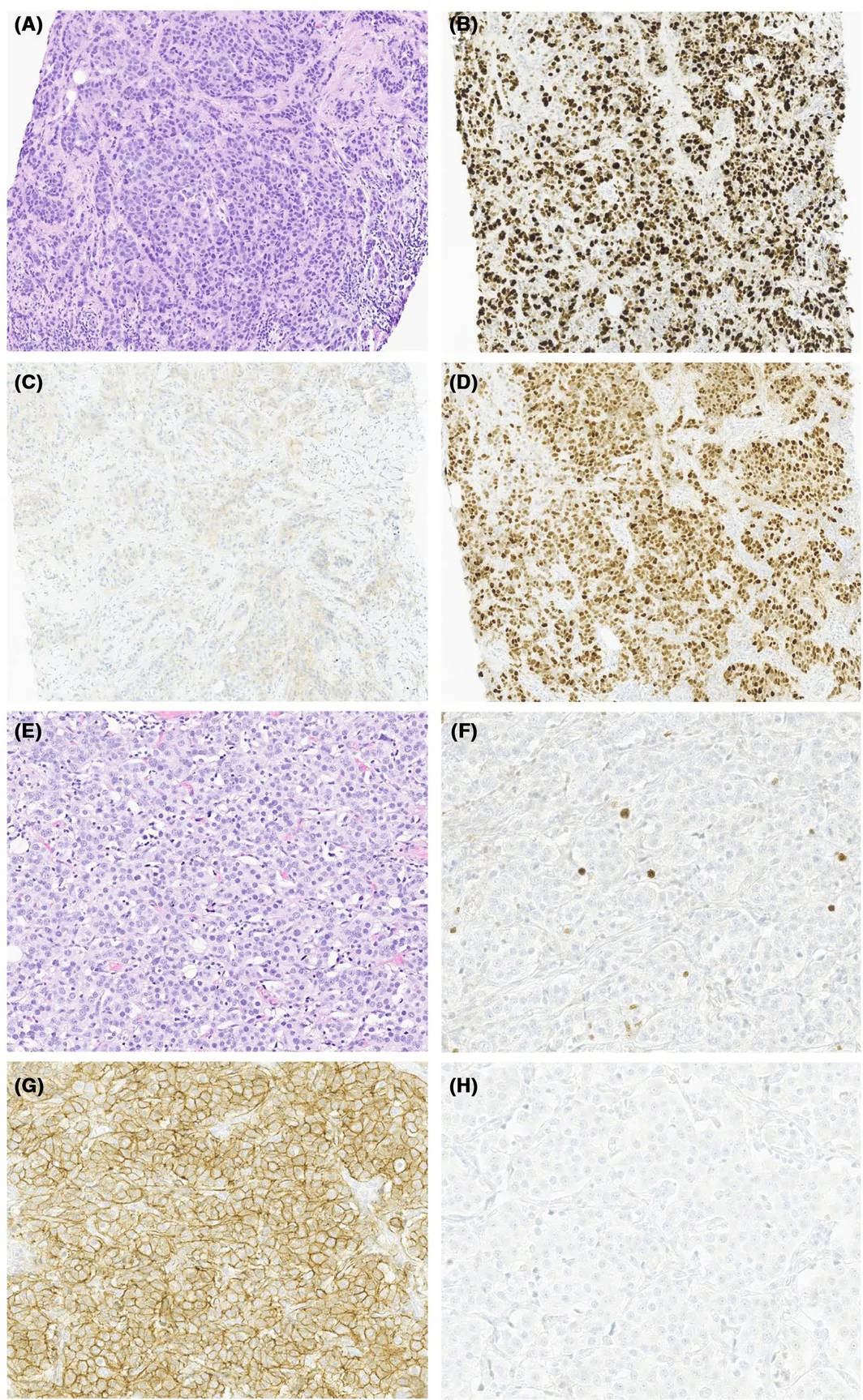
(**A**) Haematoxylin–eosin–stained pretreatment biopsy specimen of invasive breast carcinoma of no special type. (**B**) Immunohistochemistry for Ki67 showing high proliferative activity. (**C**) HER2 immunohistochemistry score 1+ in the pretreatment biopsy specimen. (**D**) Positive nuclear staining for progesterone receptor (PR) in the pretreatment biopsy specimen. (**E**) Haematoxylin–eosin–stained resection specimen after short‐course preoperative endocrine therapy. (**F**) Immunohistochemistry for Ki67 demonstrating post‐treatment suppression of proliferative activity. (**G**) HER2 immunohistochemistry score 2+ in the post‐treatment surgical specimen. HER2 in situ hybridisation demonstrating no amplification of the HER2 gene. (**H**) Loss of PR expression in the post‐treatment surgical specimen.

**Figure 2:**
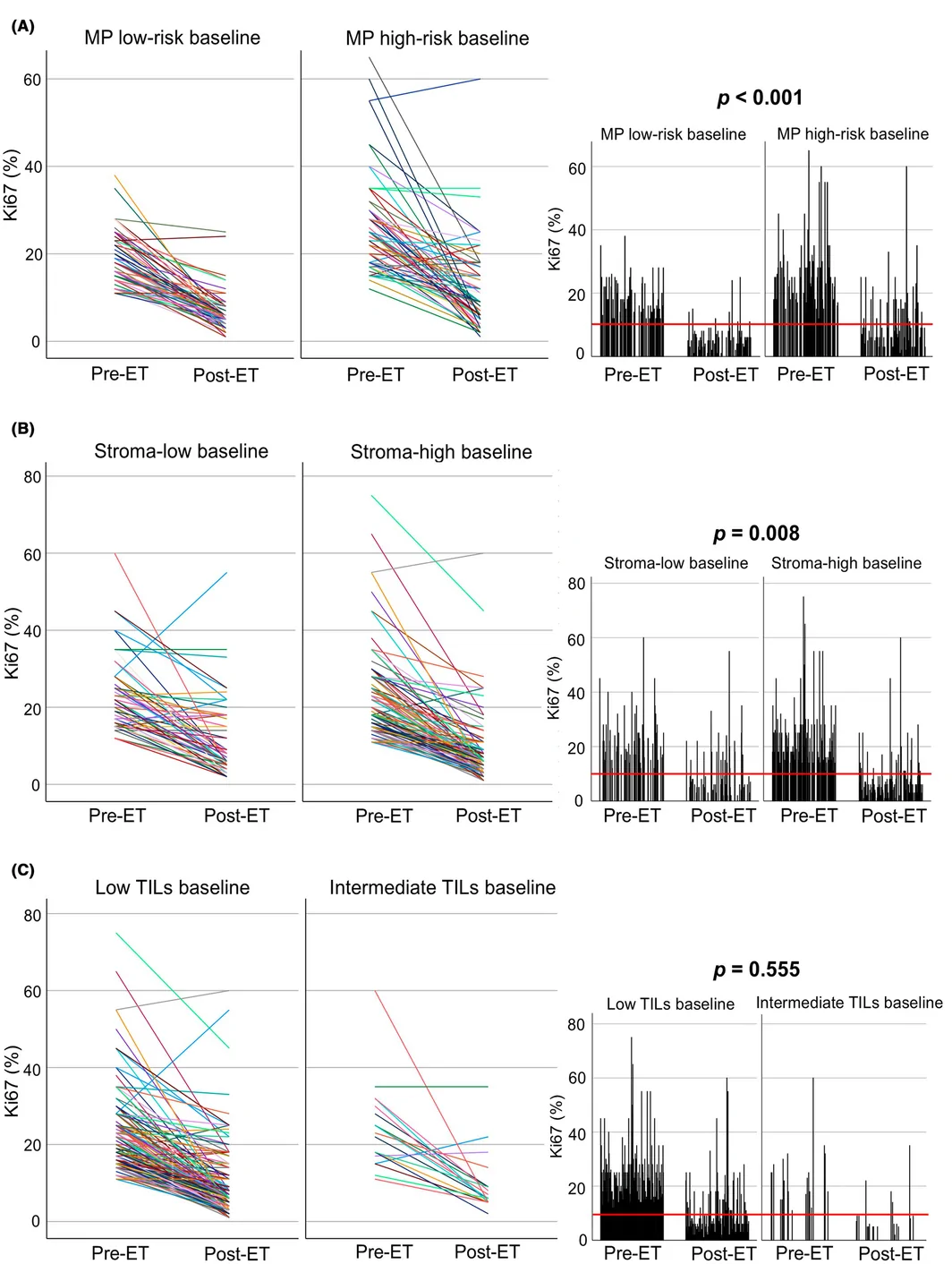
Individual changes in Ki67 from pretreatment to post‐treatment in patients treated with preoperative endocrine therapy (ET), stratified according to (**A**) baseline genomic risk by MammaPrint (MP), (**B**) baseline stromal proportion and (**C**) baseline stromal tumour‐infiltrating lymphocytes (TILs).

Short‐course preoperative ET resulted in a marked reduction in tumour proliferation (Figure 1A,B,E,F). Postsurgical Ki67 levels were significantly lower in the ET cohort compared with controls (*P* < 0.001). An endocrine response, defined as post‐treatment Ki67 ≤ 10%, was observed in 73.9% of treated patients, whereas none of the control tumours reached this threshold (*P* < 0.001) (Table 1). Median Ki67 decreased from 20% to 6% following ET, while remaining essentially unchanged in controls (median 22% to 23%). Among ET‐treated patients, responders showed a pronounced reduction in Ki67 (18% to 5%), whereas non‐responders demonstrated only a modest decrease (23% to 18%). A reduction in Ki67 of ≥10% was observed in 83.5% of responders compared with 27.7% of non‐responders (*P* < 0.001). Conversely, no significant change in Ki67 (defined as an increase or decrease of <10% relative to baseline) was seen in 16.5% of responders and 70.2% of non‐responders (*P* < 0.001) (Table 2). Response rates were higher in premenopausal compared with peri‐/postmenopausal patients (91.7% vs. 69.4%, *P* = 0.007), whereas no significant differences were observed between patients treated with AIs and those receiving tamoxifen (*P* = 0.752). Treatment duration did not correlate with post‐treatment Ki67 (*r* = 0.02, *P* = 0.803). Endocrine response was strongly associated with genomic risk. Response rates were significantly higher in low‐risk compared with high‐risk tumours (87.7% vs. 60.6%, *P* < 0.001) (Figure 2A), and the MammaPrint index showed a moderate inverse correlation with post‐treatment Ki67 (*r* = −0.37, *P* < 0.001). Median Ki67 decreased from 25% to 9% in high‐risk tumours and from 18% to 6% in low‐risk tumours.

**Table 2:** Histopathological and immunohistochemical changes in the study cohort

Variable	ET (*N* = 180)	Control (*N* = 151)	*P*‐value	ET responders (*N* = 133)	ET non‐responders (*N* = 47)	*P‐*value
	*N* (%)	*N* (%)		*N* (%)	*N* (%)	
Change in subtype						
Yes	2 (1.1)	4 (2.6)	0.296	2 (1.5)	0 (0.0)	0.398
No	178 (98.9)	147 (97.4)		131 (98.5)	47 (100.0)	
Change in grade						
Downgrading	66 (36.7)	1 (0.7)	**<0.001**	59 (44.4)	7 (14.9)	**<0.001**
Upgrading	2 (1.1)	11 (7.3)		0 (0.0)	2 (4.3)	
Stable	112 (62.2)	139 (92.1)		74 (55.6)	38 (80.9)	
Change in mitotic count						
Decrease	108 (60.0)	2 (1.3)	**<0.001**	91 (68.4)	17 (36.2)	**<0.001**
Increase	4 (2.2)	32 (21.2)		0 (0.0)	4 (8.5)	
Stable	68 (37.8)	117 (77.5)		42 (31.6)	26 (55.3)	
Change in nuclear pleomorphism						
Decrease	9 (5.0)	1 (0.7)	**0.030**	7 (5.3)	2 (4.3)	0.670
Increase	2 (1.1)	5 (3.3)		2 (1.5)	0 (0.0)	
Stable	169 (93.9)	145 (96.0)		124 (93.2)	45 (95.7)	
Change in tubule formation						
Decrease	5 (2.8)	2 (1.3)	0.067	4 (3.0)	1 (2.1)	**0.035**
Increase	23 (12.8)	9 (6.0)		22 (16.5)	1 (2.1)	
Stable	152 (84.4)	140 (92.7)		107 (80.5)	45 (95.7)	
Change in SP						
Stroma‐low → stroma‐high	19 (10.6)	13 (8.6)	0.218	12 (9.0)	7 (14.9)	0.486
Stroma‐high → stroma‐low	21 (11.7)	10 (6.6)		15 (11.3)	6 (12.8)	
Stable	140 (77.8)	128 (84.8)		106 (79.7)	34 (72.3)	
Change in TILs						
Decrease ≥10%	4 (2.2)	1 (0.7)	0.511	1 (0.8)	3 (6.4)	0.071
Increase ≥10%	6 (3.3)	5 (3.3)		5 (3.8)	1 (2.1)	
Stable	170 (94.4)	145 (96.0)		127 (95.5)	43 (91.5)	
Change in ER Allred score^a^						
Decrease	29 (16.1)	25 (16.6)	0.348	24 (18.0)	5 (10.6)	0.455
Increase	5 (2.8)	9 (6.0)		4 (3.0)	1 (2.1)	
Stable	146 (81.1)	117 (77.5)		105 (78.9)	41 (87.2)	
Change in PR status						
Yes	18 (10.0)	5 (3.3)	**0.017**	13 (9.8)	5 (10.6)	0.865
No	162 (90.0)	146 (96.7)		120 (90.2)	42 (89.4)	
Change in PR Allred score^a^						
Decrease	136 (75.6)	42 (27.8)	**<0.001**	113 (85.0)	23 (48.9)	**<0.001**
Increase	4 (2.2)	20 (13.2)		2 (1.5)	2 (4.3)	
Stable	40 (22.2)	89 (58.9)		18 (13.5)	22 (46.8)	
Change in PR status						
Biopsy+/resection +	147 (81.7)	144 (95.4)	**<0.001**	113 (85.0)	34 (72.3)	**0.025**
Biopsy +/resection ‐	17 (9.4)	2 (1.3)		13 (9.8)	4 (8.5)	
Biopsy −/resection ‐	15 (8.3)	2 (1.3)		7 (5.3)	8 (17.0)	
Biopsy −/resection +	1 (0.6)	3 (2.0)		0 (0.0)	1 (2.1)	
Change in HER2 score						
Downscoring	8 (4.4)	28 (18.5)	**<0.001**	5 (3.8)	3 (6.4)	0.755
Upscoring	47 (26.1)	10 (6.6)		35 (26.3)	12 (25.5)	
Stable	125 (69.4)	113 (74.8)		93 (69.9)	32 (68.1)	
Change in Ki67						
Decrease ≥10%	124 (68.9%)	3 (2.0)	**<0.001**	111 (83.5)	13 (27.7)	**<0.001**
Increase ≥10%	1 (0.6)	7 (4.6)		0 (0.0)	1 (2.1)	
Stable	55 (30.6)	141 (93.4)		22 (16.5)	33 (70.2)	

ER, oestrogen receptor; ET, preoperative endocrine therapy; PR, progesterone receptor; SP, stromal proportion; TILs, tumour‐infiltrating lymphocytes.

Values in bold indicate statistical significance (*P* < 0.05).

^a^ Change by at least one score.

### Histopathological Changes

**Figure 3:**
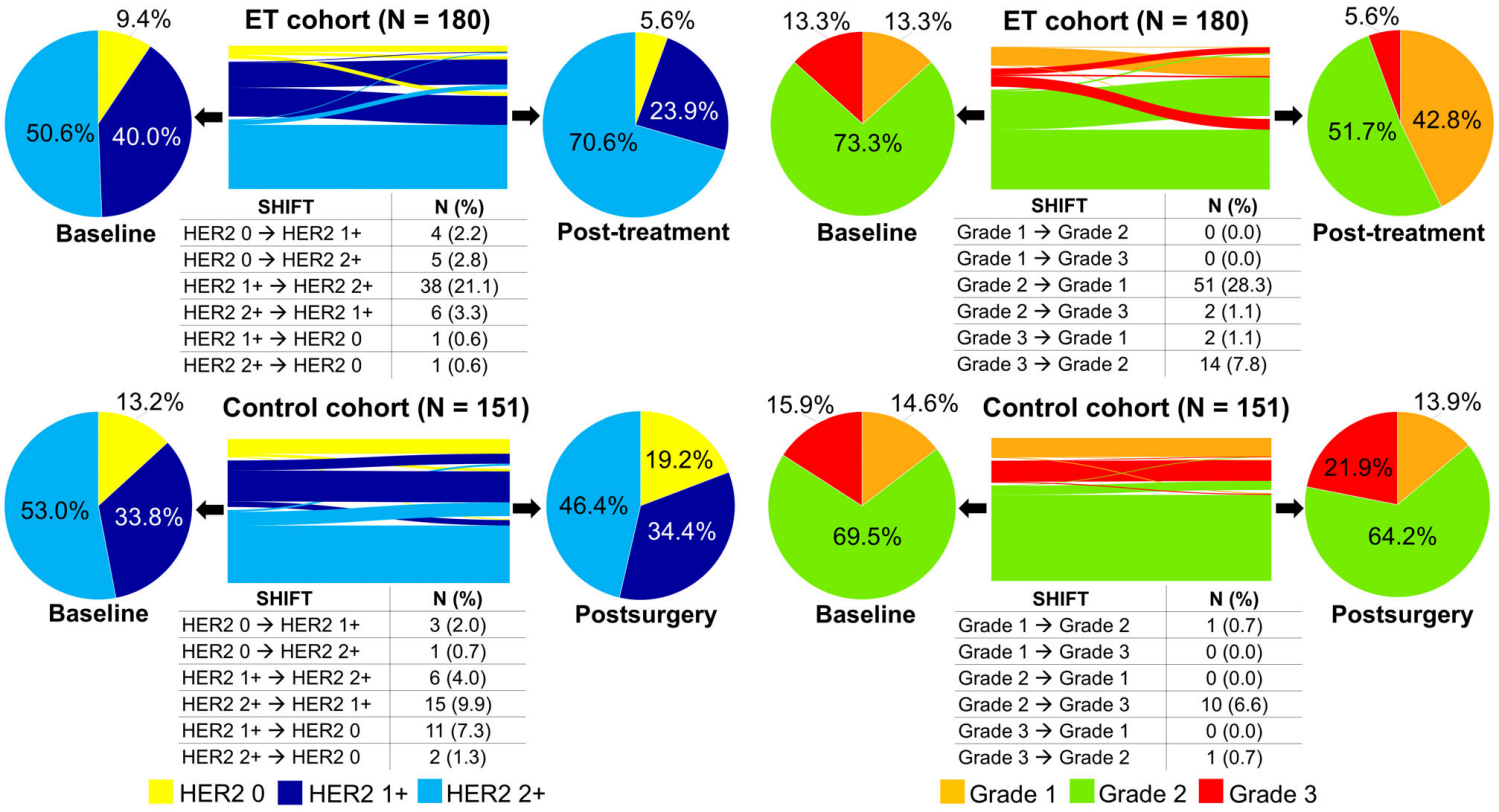
Changes in HER2 immunohistochemistry score and tumour grade in patients treated with preoperative endocrine therapy (ET) and in the control cohort.

Histological subtype remained stable between biopsy and resection specimens (Table 2, Table S1). In contrast, ET was associated with changes in tumour grade (Table 2, Table S2). Tumour downgrading was observed in 36.7% of treated cases compared with 0.7% in controls (*P* < 0.001) (Figure 3). Grade reduction was primarily driven by decreased mitotic activity, either alone (63.6%) or in combination with improved glandular differentiation and/or reduced nuclear pleomorphism, and occurred more frequently in responders than in non‐responders (44.4% vs. 14.9%, *P* < 0.001).

SP did not differ significantly between ET‐treated and control cohorts overall. However, within the ET cohort, higher baseline and post‐treatment SP were observed in responders (*P* = 0.021 and *P* = 0.007, respectively) (Table 1). Accordingly, responders more frequently exhibited a stroma‐rich phenotype (SP >50%) both at baseline and after treatment (*P* = 0.008 and *P* = 0.038) (Figure 2B). SP showed weak but significant inverse correlations with post‐treatment Ki67 (baseline: *r* = −0.26, *P* < 0.001; post‐treatment: *r* = −0.24, *P* = 0.001). Central scarring occurred at similar frequencies in both cohorts but was more common in non‐responders (*P* = 0.011) (Table 1). StrTIL levels were generally low and showed no significant association with endocrine response or Ki67 dynamics (Table 1, Figure 2C). A ≥ 10% decrease in strTILs was more frequent in non‐responders, although this did not reach statistical significance (*P* = 0.071) (Table 2).

### Immunohistochemical Changes

ER expression was retained in all cases, and changes in ER Allred score were infrequent and not associated with response. By contrast, PR expression showed marked modulation under ET. Reductions in PR Allred score and complete loss of PR expression were significantly more frequent in treated tumours than in controls (75.6% vs. 27.8% and 17.8% vs. 2.6%, respectively; both *P* < 0.001) (Figure 1D,H). Both a decrease in PR expression after treatment and higher baseline PR expression (≥50%) were significantly associated with endocrine response (*P* < 0.001 and *P* = 0.002, respectively) (Table 2). Baseline PR expression also showed a weak inverse correlation with post‐treatment Ki67 (*r* = −0.27, *P* < 0.001). ET‐treated tumours more frequently exhibited HER2‐low expression at resection compared with controls (*P* < 0.001) (Table 1, Table S3, Figure 1C,G, Figure 3). Although HER2 scores changed dynamically during treatment, these changes were not associated with Ki67‐defined response (Table 2).

### Predictors of Response

Pretreatment clinicopathological variables (Table S4), including genomic risk assessed on core biopsy specimens, were evaluated in regression models for association with endocrine response. In univariate analysis, low genomic risk, stroma‐rich phenotype, lower tumour grade (G1/G2 vs. G3), premenopausal status, younger age (≤50 years), higher baseline Ki67 and higher baseline PR Allred score were associated with response. In multivariate analysis, only genomic risk (MammaPrint) and baseline PR Allred score remained independent predictors, whereas microenvironmental parameters, including strTILs, were not significantly associated with Ki67‐defined response (Table S4).

## Discussion

The effects of preoperative ET on tumour morphology as well as immunohistochemical and biological characteristics remain incompletely defined, particularly in the context of window‐of‐opportunity approaches designed to assess endocrine sensitivity in vivo. The present study provides a detailed characterisation of histomorphological and immunophenotypic changes following short‐course preoperative endocrine induction therapy in ER‐positive, HER2‐negative BC using paired pre‐ and post‐treatment samples, a contemporaneous untreated control cohort, and integrated genomic risk assessment with MammaPrint. By applying a proliferation‐based endpoint (post‐treatment Ki67 ≤ 10%) aligned with modern response‐adapted clinical trial designs,^15^ this analysis captures early biological effects of endocrine exposure within a clinically relevant framework and links genomic risk stratification to dynamic endocrine response patterns.

Consistent with perioperative endocrine trials such as IMPACT and POETIC,^13^, ^14^ short‐course ET resulted in rapid and substantial suppression of tumour proliferation, with nearly three‐quarters of tumours achieving Ki67 ≤ 10% after approximately 4 weeks. These observations further establish early Ki67 dynamics as a robust surrogate marker of endocrine sensitivity and provide additional rationale for its integration into response‐adapted treatment strategies, as implemented in trials such as WSG‐ADAPT HR+/HER2–.^15^ Importantly, the present analysis extends these observations by demonstrating that early endocrine response is accompanied by reproducible and quantifiable histomorphological alterations. Tumour downgrading, primarily driven by reduced mitotic activity, was observed in more than one‐third of treated tumours compared with 0.7% of control cases and was strongly associated with Ki67‐defined response. These findings indicate that short‐term endocrine exposure induces a rapid cytostatic effect that is not only captured by proliferation markers such as Ki67 but also reflected in routine histopathological assessment. The predominance of these changes in responding tumours argues against mere sampling variability and underscores their biological relevance. Similar histological alterations have been reported following both prolonged NAET^9^, ^10^, ^11^, ^12^ and short‐course bridging NAET administered during the COVID‐19 pandemic,^5^ with reported downgrading rates ranging from 12.1%^5^ to 30.4%.^9^ However, these bridging NAET studies, including the multicentre analysis by Miligy *et al*.,^5^ differed substantially from the present analysis in terms of treatment intent, patient selection, duration of therapy and endpoint definition, as they primarily reflected pragmatic surgical delay strategies rather than biologically driven response‐adapted treatment approaches. Notably, in the present study, morphologically appreciable treatment effects were already detectable after brief endocrine exposure, highlighting the sensitivity of early response assessment in luminal BC.

At the immunophenotypic level, modulation of PR expression emerged as a key feature of early endocrine response. Both baseline PR expression and treatment‐related reduction in PR levels were strongly associated with proliferative suppression, consistent with the role of PR as a downstream target of ER signalling.^37^ Together, these observations suggest that changes in PR expression may serve as a pharmacodynamic marker of effective pathway inhibition^38^ and highlight its potential utility as a clinically accessible biomarker complementary to Ki67 in response‐adapted strategies. By contrast, ER expression remained largely stable, underscoring distinct regulatory mechanisms during ET and limiting its utility as a dynamic response marker. Changes in ER and PR expression have previously been described following NACT and, to a lesser extent, NAET.^5^, ^9^, ^39^, ^40^, ^41^, ^42^ Badr *et al*.^9^ reported changes in PR Allred scores between pretreatment and post‐treatment specimens in 52.2% of cases, with conversion from PR‐positive to PR‐negative status in 17.6% after 6 months of NAET. Similarly, Miligy *et al*.^5^ observed loss of PR expression in 23.1% of tumours after a median treatment duration of 45 days of bridging NAET during the COVID‐19 pandemic, suggesting that PR modulation may occur even after relatively short endocrine exposure. By contrast, consistent with our findings demonstrating retained ER expression in all post‐treatment tumour samples, conversion from ER‐positive to ER‐negative status appears to be rare following both prolonged NAET (0.7%)^9^ and shorter bridging NAET approaches (0.8%).^5^

We also observed an increased frequency of HER2‐low expression after short‐course preoperative endocrine induction therapy. Although changes in HER2 immunohistochemical score were not associated with Ki67‐defined response, the observed shift toward increased HER2‐low expression may reflect treatment‐related modulation of protein expression. Similar dynamics have been reported previously. For instance, Badr *et al*.^9^ described conversion from HER2‐negative to HER2‐positive status in a small subset of cases (6.1%) following prolonged NAET, whereas conversion from HER2‐positive to HER2‐negative status was infrequent (3.8%). In a comparative analysis of paired tumour samples, Schettini *et al*.^43^ observed frequent shifts between HER2‐zero and HER2‐low categories in patients with HR‐positive, HER2‐negative disease treated with either NACT or 3–6 months of NAET, although conversion to HER2‐positive status occurred exclusively in the chemotherapy‐treated cohort. Miligy *et al*.^5^ similarly reported post‐treatment changes in HER2 status in both directions, albeit at low frequency (up to 3.5%), together with conversion from HER2‐low to HER2‐zero expression in approximately one‐third of cases following short‐course bridging NAET. Collectively, these findings suggest that both treatment modality and duration may differentially influence HER2 expression patterns. The mechanisms underlying these changes remain incompletely understood but may involve adaptive transcriptional plasticity and compensatory signalling responses under therapeutic pressure.^44^, ^45^, ^46^, ^47^ The current results further underscore the dynamic nature of HER2 expression, which may be modulated even after relatively brief endocrine exposure. Given the increasing therapeutic relevance of HER2‐low disease, particularly in the context of antibody–drug conjugates, reassessment of HER2 status following short‐course preoperative endocrine induction therapy may therefore be clinically informative, even in tumours initially classified as HER2‐negative.

In contrast to tumour‐intrinsic features, tumour microenvironmental characteristics appeared to play a limited role in predicting Ki67‐defined response. StrTIL levels were generally low in both biopsy and resection specimens and were not significantly associated with Ki67 suppression following endocrine induction therapy. The role of strTILs in the setting of short‐course preoperative ET has not yet been systematically characterised histopathologically. Available data regarding strTIL dynamics during ET are heterogeneous and largely derived from studies of prolonged treatment, which differ from short‐course approaches with respect to duration, therapeutic intent and endpoint definition, frequently relying on size‐based or objective response criteria.^32^, ^48^, ^49^ While some studies have reported treatment‐associated increases in strTIL levels, particularly in non‐responding tumours,^32^ others have observed increases in responders or largely stable levels irrespective of response status.^48^, ^49^ Collectively, these findings indicate that the relationship between immune infiltration and endocrine sensitivity is complex and context‐dependent. The results of the present study suggest that immune infiltration in luminal BC remains relatively stable during short‐term endocrine exposure and may therefore have limited relevance as a determinant of early endocrine response in this setting. Similarly, SP showed only weak associations with Ki67‐defined response. Although stroma‐rich tumours were somewhat more frequent among responders, the biological significance of this observation remains uncertain and may reflect complex tumour–stroma interactions rather than a direct correlate of endocrine sensitivity. Central scarring, previously described as a feature of endocrine treatment effect following both prolonged NAET^9^, ^11^ and shorter pandemic‐related bridging NAET,^5^ was observed at comparable frequency in ET‐treated and control cohorts and was more common in non‐responders, arguing against its role as a specific marker of treatment efficacy in the short‐course setting.

A central finding of this study is the strong association between genomic risk and endocrine responsiveness. MammaPrint effectively stratified tumours according to the likelihood of Ki67 suppression, with high‐risk tumours showing significantly lower rates of post‐treatment Ki67 ≤ 10%. Consistent with their underlying biology, these tumours, characterised by increased proliferative drive and reduced dependence on ER signalling,^19^, ^20^, ^21^, ^36^, ^50^, ^51^ appear less susceptible to short‐term endocrine deprivation. Activation of alternative proliferative pathways, including PI3K/AKT/mTOR and cyclin D–CDK4/6 signalling, may further sustain tumour growth despite ER blockade.^52^, ^53^ These findings are concordant with prospective data, including the WSG‐ADAPT trial,^15^ linking genomic risk to endocrine sensitivity, and extend this concept by demonstrating corresponding morphological and immunophenotypic correlates. Thus, genomic risk appears not only prognostic but also biologically reflected in distinct patterns of early treatment‐induced tumour remodelling.

From a clinical perspective, our results support the use of short‐course preoperative ET as an in vivo functional assay of tumour biology. The integration of genomic risk assessment, as established in the MINDACT trial,^19^, ^21^ with dynamic biomarkers such as Ki67 and PR expression may provide a clinically applicable framework for treatment stratification. Patients with genomically low‐risk tumours and early endocrine response may represent candidates for treatment de‐escalation, whereas those with high‐risk or endocrine‐resistant disease may benefit from alternative or intensified therapeutic approaches. Importantly, the present study adds a pathological dimension to these clinically driven concepts by defining the histomorphological and immunophenotypic features associated with early endocrine response. This has practical implications for diagnostic pathology, as awareness of therapy‐induced histopathological changes is essential to ensure accurate interpretation of post‐treatment specimens in routine practice and to avoid misclassification.

Several limitations should be acknowledged, including the retrospective single‐centre design and incomplete availability of genomic data. In addition, Ki67 assessment is inherently subject to interobserver variability, although standardised evaluation criteria were applied. Furthermore, long‐term clinical outcome data were not available, precluding direct correlation between early biological response and survival outcomes. Despite these limitations, the study is strengthened by centralised pathology review, paired sample analysis and the inclusion of a contemporaneous control cohort, enabling robust differentiation between treatment‐related effects and temporal or sampling‐related variability.

## Conclusions

Short‐course preoperative ET induces rapid and reproducible morphological and immunophenotypic changes in ER‐positive, HER2‐negative BC. While MammaPrint is an established predictor of endocrine sensitivity, our findings suggest that genomic risk is associated with distinct post‐treatment patterns of tumour differentiation and biomarker modulation, linking molecular risk stratification with phenotypic endocrine response. Together, these results support the integration of genomic and dynamic biomarkers in response‐adapted treatment strategies and highlight the expanding role of pathology in precision oncology.

## Author contributions

C.G. designed the study, collected and analysed the clinicopathological data, performed the statistical analysis and drafted the manuscript. A.G. and P.N. interpreted the data and revised the manuscript. C.I.P., H.K.S., B.S., T.G. and P.S. contributed to data acquisition and revised the manuscript. R.L. supervised the study and critically revised the manuscript.

## Funding information

The authors have nothing to report.

## Conflict of interest

The authors have no conflicting interests to disclose.

## Supporting information


**Table S1.** Histological tumour subtypes in the ET and control cohorts.
**Table S2**. Tumour grade in the ET and control cohorts.
**Table S3**. HER2 scores in the ET and control cohorts.
**Table S4**. Univariate and multivariate analysis for Ki67‐defined response in ET‐treated patients.

## Data Availability

The data supporting the findings of this study are available from the corresponding author upon reasonable request.
