# Supplementary material for: Effects of short‐course preoperative endocrine therapy on tumour morphology and immunohistochemical profile in oestrogen receptor‐positive, HER2‐negative breast cancer
Source: Histopathology. 2026 Jun 18;89(4):670–86. doi: 10.1111/his.70203 (PMC13551165; doi:10.1111/his.70203)
Supplement: Supplementary file 1 [file HIS-89-670-s001.docx]

**Table S1. Histological tumour subtypes in the ET and control cohorts.**

| **ET cohort** | **Subtype post-treatment** | | | |  |  |  |
| --- | --- | --- | --- | --- | --- | --- | --- |
| **Subtype baseline** | NST | Lobular | Mixed ductal/lobular | Mucinous | Micropapillary | Mixed (NST+ST) | Total |
| NST | 133 | 0 | 0 | 0 | 0 | 1 | 134 |
| Lobular | 0 | 24 | 0 | 0 | 0 | 0 | 24 |
| Mixed ductal/lobular | 0 | 0 | 4 | 0 | 0 | 0 | 4 |
| Mucinous | 0 | 0 | 0 | 3 | 0 | 0 | 3 |
| Micropapillary | 0 | 0 | 0 | 0 | 3 | 0 | 3 |
| Mixed (NST+ST) | 0 | 0 | 0 | 1 | 0 | 11 | 12 |
| Total | 133 | 24 | 4 | 4 | 3 | 12 | 180 |
| **Control cohort** | **Subtype postsurgical** | | | |  |  |  |
| **Subtype baseline** | NST | Lobular | Mixed ductal/lobular | Mucinous | Micropapillary | Mixed (NST+ST) | Total |
| NST | 119 | 0 | 1 | 0 | 0 | 2 | 122 |
| Lobular | 0 | 12 | 0 | 0 | 0 | 0 | 12 |
| Mixed ductal/lobular | 0 | 0 | 1 | 0 | 0 | 0 | 1 |
| Mucinous | 0 | 0 | 0 | 5 | 0 | 0 | 5 |
| Micropapillary | 0 | 0 | 0 | 0 | 0 | 1 | 1 |
| Mixed (NST+ST) | 0 | 0 | 0 | 0 | 0 | 10 | 10 |
| Total | 119 | 12 | 2 | 5 | 0 | 13 | 151 |

ET, preoperative endocrine therapy; NST, no special type; ST special type.

**Table S2. Tumour grade in the ET and control cohorts.**

| **ET cohort** | **Grade post-treatment** | | | |
| --- | --- | --- | --- | --- |
| **Grade baseline** | 1 | 2 | 3 | Total |
| 1 | 24 | 0 | 0 | 24 |
| 2 | 51 | 79 | 2 | 132 |
| 3 | 2 | 14 | 8 | 24 |
| Total | 77 | 93 | 10 | 180 |
| **Control cohort** | **Grade postsurgical** | | | |
| **Grade baseline** | 1 | 2 | 3 | Total |
| 1 | 21 | 1 | 0 | 22 |
| 2 | 0 | 95 | 10 | 105 |
| 3 | 0 | 1 | 23 | 24 |
| Total | 21 | 97 | 33 | 151 |

ET, preoperative endocrine therapy.

**Table S3. HER2 scores in the ET and control cohorts.**

| **ET cohort** | **HER2 post-treatment** | | | |
| --- | --- | --- | --- | --- |
| **HER2 baseline** | 0/ultra-low | 1+ | 2+ | Total |
| 0/ultra-low | 8 | 4 | 5 | 17 |
| 1+ | 1 | 33 | 38 | 72 |
| 2+ | 1 | 6 | 84 | 91 |
| Total | 10 | 43 | 127 | 180 |
| **Control cohort** | **HER2 postsurgical** | | | |
| **HER2 baseline** | 0/ultra-low | 1+ | 2+ | Total |
| 0/ultra-low | 16 | 3 | 1 | 20 |
| 1+ | 11 | 34 | 6 | 51 |
| 2+ | 2 | 15 | 63 | 80 |
| Total | 29 | 52 | 70 | 151 |

ET, preoperative endocrine therapy.

**Table S4. Univariate and multivariate analysis for Ki67-defined response in ET-treated patients.**

| **Univariate** |  | | |
| --- | --- | --- | --- |
| **Variables** | **Odds ratio** | **95% CI** | ***p*-Value** |
| Age (years) |  |  |  |
| ≤ 50 | 1.00 |  | **0.020** |
| > 50 | 0.27 | 0.09-0.81 |  |
| Menopausal status |  |  |  |
| Premenopausal | 1.00 |  | **0.012** |
| Peri-/postmenopausal | 0.21 | 0.06-0.71 |  |
| Tumour size (mm) |  |  |  |
| < 20 | 1.00 |  | 0.380 |
| ≥ 20 | 0.74 | 0.37-1.46 |  |
| Subtype |  |  |  |
| No special type | 1.00 |  | 0.916 |
| Other | 1.04 | 0.49-2.23 |  |
| Grade baseline |  |  |  |
| G1/G2 | 1.00 |  | **0.022** |
| G3 | 0.36 | 0.15-0.86 |  |
| SP baseline (%) |  |  |  |
| Stroma-low (≤ 50) | 1.00 |  | **0.009** |
| Stroma-high (> 50) | 2.49 | 1.25-4.94 |  |
| TILs baseline (%) |  |  |  |
| Low TILs (0-9) | 1.00 |  | 0.557 |
| Intermediate TILs (10-59) | 0.69 | 0.20-2.40 |  |
| MammaPrint baseline |  |  |  |
| Low-risk | 1.00 |  | **<0.001** |
| High-risk | 0.22 | 0.09-0.52 |  |
| HER2 baseline |  |  |  |
| Score 0 | 1.00 |  | 0.648 |
| Score 1+ | 1.31 | 0.41-4.12 | 0.602 |
| Score 2+ | 1.21 | 0.60-2.44 |  |
| Ki67 baseline (%) | 1.05 | 1.02-1.08 | **0.003** |
| PR Allred score baseline | 0.81 | 0.71-0.93 | **0.002** |
| **Multivariate** |  |  |  |
| **Variables** | **Odds ratio** | **95% CI** | ***p*-Value** |
| Age (years) |  |  |  |
| ≤ 50 | 1.00 |  | 0.764 |
| > 50 | 1.36 | 0.18-10.21 |  |
| Menopausal status |  |  |  |
| Premenopausal | 1.00 |  | 0.051 |
| Peri-/postmenopausal | 0.10 | 0.01-1.01 |  |
| Grade biopsy |  |  |  |
| G1/G2 | 1.00 |  | 0.706 |
| G3 | 1.30 | 0.34-5.01 |  |
| SP baseline (%) |  |  |  |
| Stroma-low (≤ 50) | 1.00 |  | 0.369 |
| Stroma-high (> 50) | 1.52 | 0.61-3.82 |  |
| MammaPrint baseline |  |  |  |
| Low-risk | 1.00 |  | **0.002** |
| High-risk | 0.20 | 0.07-0.57 |  |
| Ki67 baseline (%) | 1.00 | 0.95-1.06 | 0.929 |
| PR Allred score baseline | 0.82 | 0.69-0.99 | **0.038** |

ET, preoperative endocrine therapy; SP, stromal proportion; TILs tumour-infiltrating lymphocytes, PR, progesterone receptor.
